# Live Cell Characterization of DNA Aggregation Delivered through Lipofection

**DOI:** 10.1038/srep10528

**Published:** 2015-05-27

**Authors:** Stephen Mieruszynski, Candida Briggs, Michelle A. Digman, Enrico Gratton, Mark R Jones

**Affiliations:** 1 University of Western Sydney, School of Science and Health, Hawkesbury Campus, Locked Bag 1797, Penrith NSW 2751, Australia; 2 Department of Developmental and Cell Biology, University of California Irvine, Irvine, California, United States of America; Department of Biomedical Engineering, Laboratory for Fluorescence Dynamics, University of California Irvine, Irvine, California, United States of America; 3 Centre for Bioactive Discovery in Health and Ageing, School of Science & Technology, University of New England, Armidale, Australia

## Abstract

DNA trafficking phenomena, such as information on where and to what extent DNA aggregation occurs, have yet to be fully characterised in the live cell. Here we characterise the aggregation of DNA when delivered through lipofection by applying the Number and Brightness (N&B) approach. The N&B analysis demonstrates extensive aggregation throughout the live cell with DNA clusters in the extremity of the cell and peri-nuclear areas. Once within the nucleus aggregation had decreased 3-fold. In addition, we show that increasing serum concentration of cell media results in greater cytoplasmic aggregation. Further, the effects of the DNA fragment size on aggregation was explored, where larger DNA constructs exhibited less aggregation. This study demonstrates the first quantification of DNA aggregation when delivered through lipofection in live cells. In addition, this study has presents a model for alternative uses of this imaging approach, which was originally developed to study protein oligomerization and aggregation.

In gene therapy, the delivery of nucleic acids involves the coupling of the nucleic acid, such as DNA, with a vector. This facilitates the cellular uptake and subsequent processing. A common approach, utilized by many research groups, is the use of lipids. Lipoplexes are formed between the lipid and the DNA utilizing temporary electrostatic forces^1^. Lipofection offers many advantages over other approaches, i.e. the use of viruses, but lacks the efficiency of other delivery mechanisms^2^.

At present, many processes involved in lipid-based gene delivery have been well researched and documented to achieve clinically relevant outcomes. In the first instance, the lipoplex is expected to first enter the cell via endocytosis^3^ and then traffic through the cytoplasm along the microtubule network^4^. At the same time, the lipoplexes face a reduced motion within the cytoplasm^5^. Eventually, the delivered DNA is expected to enter the nucleus through nuclear pore complexes^6^,^7^, or associates with nuclear components during cell division^8^,^9^.

However, during the DNA delivery process the aggregation of the lipoplexes within the live cell milieu has not been characterised. Aggregation of the delivered DNA and lipoplex is likely to pose a significant mechanical and physical barrier. A key limitation hampering the study of aggregation has been the technological difficulty to quantify aggregation in live cells. The recently developed bioimaging tool Number and Brightness (N&B) previously utilised to investigate protein aggregation and stoichiometry in living cells^10^,^11^,^12^,^13^,^14^,^15^, which can now be applied to study DNA aggregation.

The N&B approach works on the principles of Fluorescence Correlation Spectroscopy (FCS). The particle of interest must be fluorescently labelled and upon focusing a laser source onto the sample, an illumination volume is created. Within the sample, particles are expected to move through the illumination volume over time, producing fluctuations. Based on the variances in intensity of these fluctuations the aggregative state can be elucidated. After acquiring an image series, the apparent brightness (B) and apparent number (N) are calculated through algorithms previously published^11^,^12^. Thus an oligomer will be differentiated from a monomeric particle by the increased brightness (B). In addition, the N&B approach presents the number and brightness data as a series of maps and histograms, enabling regions of aggregation in the cell to be identified with a single pixel resolution^12^.

Therefore, in this study we have applied the N&B approach to quantity lipoplex aggregation in live cells^11^,^12^. In our study, we first demonstrate that the N&B technique is able to determine DNA aggregation, and then apply the approach to characterise DNA/lipoplex aggregation through the live cell. For our model, the myoblast cell line was utilised, since muscle is an ideal gene therapy target for transgene expression and secretion. We then explore the changes in aggregation due to the serum conditions in culture, and the effects of DNA size. Here the N&B approach was applied to investigate various sized DNA rather than expressed GFP-tagged proteins, demonstrating differences in aggregation due to location and cell behaviour.

## Results

### The Number and Molecular Brightness Approach to Quantify Aggregation

**Figure 1:**
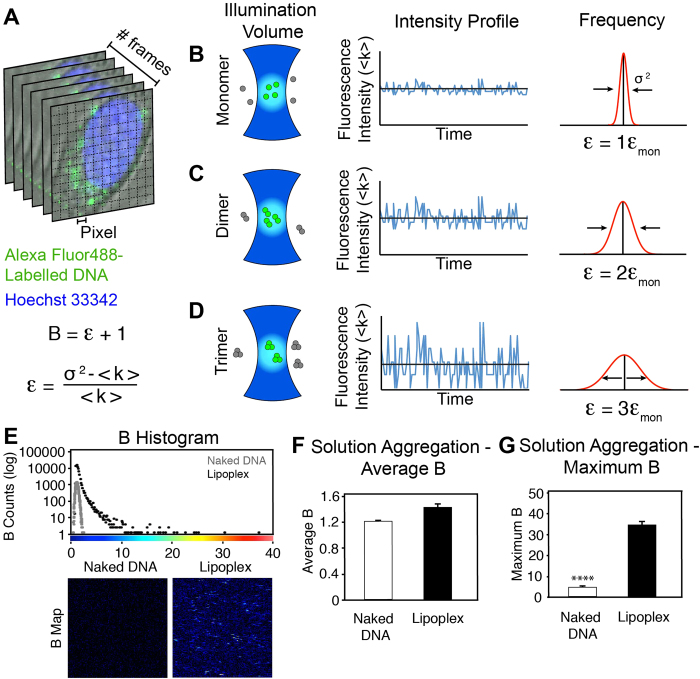
The N&B Method to Quantify Lipoplex Aggregation. (**A**) Typical image stack consisting of 100 sequential frames. Hoechst 33342 labels the nucleus whilst Alexa Fluor488 labels the introduced DNA complexes. (**B**–**D**) Schemetic of theoretical conditions within an illumination volume: (**B**) monomer, (**C**) dimer and (**D**) trimer (aggregative states). In the illumination volume, or PSF, fluorescently labelled particles e.g. lipoplexes, will move through the volume resulting in fluctuations over time (adjacent intensity profiles) for each pixel. In the case of a monomer, fluctuations will be minor over time compared to the much broader fluctuations as the molecules aggregate. Through the analysis of the distribution of the fluctuations, the variance (σ^2^) is obtained, which is directly related to the aggregative state. Mathematically, the aggregation is represented as the apparent brightness (**B**), which can be used to calculate the true molecular brightness (ε), which takes into account the variance and average intensity (<k>) (equations presented). As a molecule aggregates and the fluctuations broaden, ε will increase proportional to the aggregation state; for example in a dimer ε will be double that of a monomer, or triple in a trimer. In addition, the ratio of <k> squared and σ^2^, is proportional to the average particle number, which can also be determined. The illustration was adapted from Presman *et al.* (2014)^22^. (**E**) As a proof-of-concept, DNA was studied in solution, presented as a B histogram and B maps. The B maps were colour coded based on the colour palette along the histogram x-axis. The maximum extend of aggregation was 8-fold greater in the lipoplexes, whereas the average B was similar (**F**, **G**). Graphs depict mean ± se. **** p < 0.0001.

To quantify the aggregation of delivered DNA and lipoplexes the N&B approach was applied. This technique is based on the moment analysis of intensity fluctuations at a pixel level, which provides details on the aggregative state and particle number in an image series^11^,^12^. In this approach, an oligomer will show as a particle of brightness (B) n-times the brightness of a monomeric particle. Data is presented in a series of maps, plots and histograms enabling the spatial quantification of aggregation (Fig. 1A–D).

The N&B approach is a valuable tool to assess DNA and lipoplex aggregation. Firstly, it can be seen that the DNA alone in solution did not aggregate (Fig. 1E). Once the DNA lipoplexes were formed, aggregation was revealed by an 8-fold increase in the highest B values obtained (Fig. 1G). Secondly, when DNA and lipoplexes are in solution, the average B values obtained were similar to naked DNA in a homogenous solution. Furthermore, since the lipoplexes formed few, but large aggregates, a major portion of the area possessed no signal and this resulted in a lower average (Fig. 1F).

### Determining Aggregation in Live Cells using N&B

**Figure 2:**
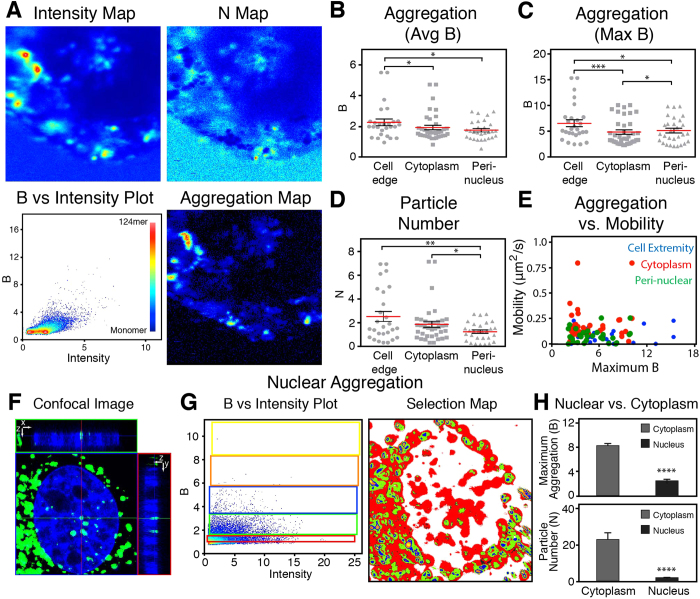
Characterizing the Intercellular Aggregation of Lipoplexes. Myoblast cells were transfected with an Alexa Fluor488 labelled DNA plasmid facilitated by lipofection. (**A**) N&B analysis 6 h after the administration of lipoplexes. A region-of-interest (ROI) analysis was performed highlighting the cell edge (circles), cytoplasm (squares) and peri-nuclear region (triangles). (**B**–**D**) Shows the type of aggregation of particles at cell edge, cytoplasm and perinuclear regions using average brightness (2B), maximum B (2C) and particle number (2D). (**E**) Influence of aggregation on the mobility of the DNA (n = 12 cells for ROI analysis). (**F**) Confocal 3D image with orthogonal views depicting the localization of the fluorescently-labelled DNA (green) in the nucleus (blue, Hoechst 33342) after 36 h. (**G**) N&B analysis of the nuclear localised DNA, presented through a B vs Intensity plot, and selection map (monomers, 2–12 mers, 12–25 mers, 25–40 mers and 40–58 mers (red, green, blue, orange and yellow, respectively). (**H**) Comparison of the aggregation and particle number of the nucleus and cytoplasm (n = 6 cells for nuclear localisation). Image size 12.8 × 12.8 μm. Graphs depict mean ± se. * p ≤ 0.05, ** p ≤ 0.01, *** p ≤ 0.001, **** p < 0.0001.

Six hours after administration of the DNA lipoplexes to live cells from our model myoblast cell line, a signal for the fluorescently labelled DNA was observed throughout the cell (Fig. 2A). The N&B map series showed an accumulation of the DNA along the extremity or edge of the cell, and the presence of DNA throughout the rest of the cell cytoplasm (Fig. 2A). DNA aggregation was quantified through a B vs. Intensity plot. Here each pixel has a position in the plot relative to the aggregative state. An increase in the B value is indicative of an increase in aggregation. In the cell presented, aggregation was found throughout the cell wherever the DNA was present. Clusters with high aggregative states (i.e. with a B value greater than 6) were found along the extremity of the cell, and in the peri-nuclear area. The highest aggregative state found in the cell was aggregates containing up to 124 DNA particles (124 mer) (monomer: 1080 cpsm, 124 mer: 133,900 cpsm) found in the peri-nuclear area (Fig. 2A). However, the large extents of aggregation observed were not commonly present. For instance, in the cell presented in Fig. 2A, pixels with a B value greater than 10 (80 mers) only accounted for 0.4% pixels of the image. A more detailed analysis of the cell in Fig. 2A has been presented in Supplementary Figure 1.

Region-of-Interest (ROI) analysis was performed on the population of cells transfected with the plasmid DNA between 4–8 h, thus providing details on small areas within the cell 1.6 × 1.6 μm in size with a pixel size of 50 nm. A trend in the aggregation and particle number, based on the location in the cell, was revealed (Fig. 2B–D). The greatest extent of aggregation was observed along the cell extremity, followed by the peri-nuclear regions, and least amount of aggregation was determined in the cytoplasm (Fig. 2C). In addition, the N&B analysis provided details on the particle number of DNA in these areas. The particle number decreased further into the cell, from the plasma membrane, containing the greatest, to the peri-nuclear region, exhibiting the least (Fig. 2D).

Once within the cell a range of aggregation was observed resulting in a large variance between cells and different locations within a single cell. In Fig. 2C for example, along the cell edge, B values ranged 2.4–15.3. As seen in Fig. 2A, the DNA within the cell is maintained in clusters containing different states of aggregation. It appears that the delivered DNA is not maintained in a single aggregative state, resulting in the wide variance.

During the ROI analysis, the Raster Image Correlation Spectroscopy (RICS) approach was also applied, providing a quantitative insight into the mobility of particles in an area^16^. RICS demonstrated that the DNA aggregation influenced the mobility of the DNA. As the aggregation increased, the rate of motion decreased in throughout the cell: along the cell edge, cytoplasm and peri-nuclear areas (Fig. 2E).

Following incubation, aggregation of the delivered DNA was observed within the nucleus (Fig. 2F). Thirty-six hours after the addition of the lipoplexes, the aggregation between the cytoplasm and the nucleus were compared. In the nucleus, the aggregative state did not exceed a B value of 5.7 (45 mers) compared to the cytoplasm, where B values of up to 12.3 (97 mers) were observed. Fig. 2G depicts the B vs Intensity plot, and selection map, in which cursors select areas of the plot (i.e. extents of aggregation) and the pixels are highlighted in the selection map (colouring of Fig. 2G: monomers, 2–12 mers, 12–25 mers, 25–40 mers and 40–58 mers (red, green, blue, orange and yellow, respectively)). In the cells where nuclear localisation was observed, the N&B analysis demonstrated a 3-fold difference between the nucleus and observable cytoplasm (Fig. 2H). In addition, the particle number within the nucleus appeared to be 90% less than the cytoplasm (Fig. 2H).

### Effects of Serum on Cytoplasmic Aggregation

**Figure 3:**
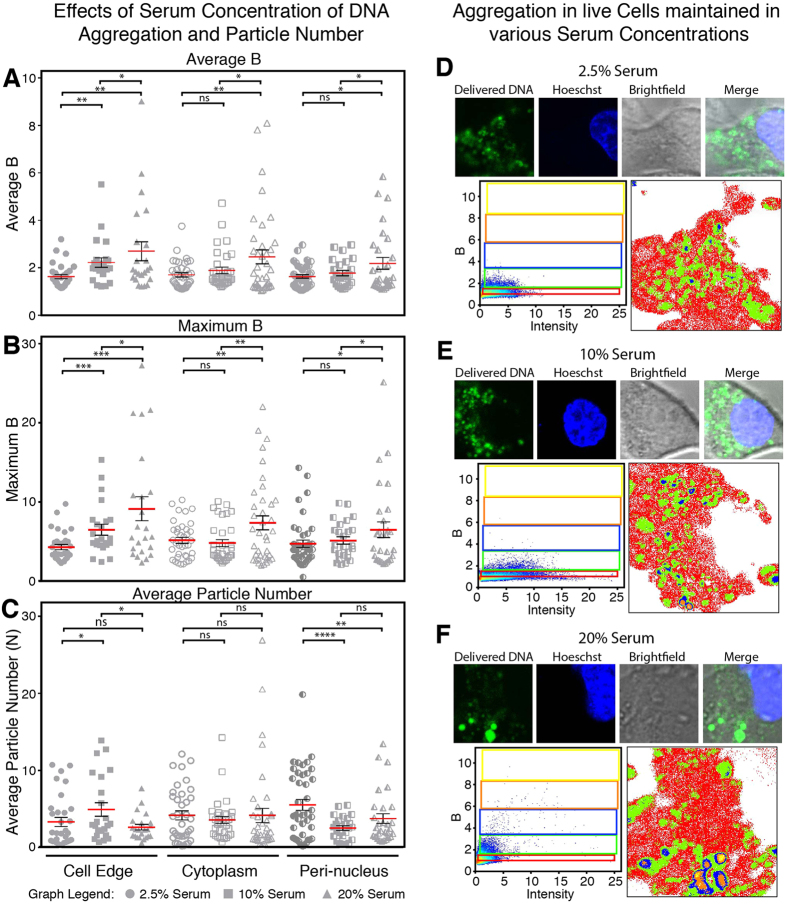
Effects of Serum Amounts on Cytoplasmic Aggregation. The L6 myoblasts were maintained and transfected in different serum (FBS) (2.5, 10 or 20% v/v) concentrations to explore to effects on cytoplasmic aggregation. (**A**–**C**) ROI analysis of DNA aggregation and particle number along the cell edge, cytoplasm and peri-nuclear region in different serum concentrations. Circles, squares and triangle show the effect of 2.5%, 10% and 20% serum respectively. (**C**–**F**) Cells transfected and maintained in 2.5% (**C**), 10% (**D**) and 20% (**F**) serum showing DNA localization and aggregation. Aggregation is presented at 5 extents: monomers, 2–12 mers, 12–25 mers, 25–40 mers and 40–58 mers (red, green, blue, orange and yellow, respectively). Each cell has been presented with a confocal image and associated N&B analysis. Supplementary Figure 3 presents the B histograms for the cells **D**–**F**. Image size 12.8 × 12.8 μm. Graphs depict mean ± se. ns = no significantly difference, * p ≤ 0.05, ** p ≤ 0.01, *** p ≤ 0.001, **** p < 0.0001.

The effect of serum concentrations (2.5, 10 and 20%) on the intracellular aggregation of DNA and lipoplexes was also explored. A Region-of-Interest (ROI) analysis demonstrates a similar trend in the control cell population as before (Fig. 2B–D). The delivered DNA was distributed throughout the cell (Fig. 3D), with the greatest extent of aggregation observed along the cell cytoplasmic edge and the lowest amount of aggregation in the cytoplasm (Fig. 3B, circles). The DNA particle number was also the greatest along the cell extremity (N = 2.5), which had dropped by 28% in the cytoplasm (N = 1.8) and was half within the peri-nuclear area compared to the cell edge (N = 1.3) (Fig. 3C, circles).

When starved and maintained in 2.5% serum, a decrease in aggregation was observed in all three locations compared to the control (Fig. 3E). In the average brightness, the most significant change occurred along the edge of the cell with a decrease of 28% (Fig. 3A, B, squares). Within the peri-nuclear region however, the highest extent of aggregation observed was 50% higher than the control (B = 14.3 compared to 9.8) (Fig. 3B, squares). When starved, the DNA particle number demonstrated the least amount of DNA along the cell edge, and the greatest particle number in the peri-nuclear region. Within the cytoplasm a similar amount was observed compared to the control (Fig. 3C, squares). The distribution of DNA particle number in the starved condition was opposite to the control.

The cells maintained in 20% serum demonstrated fewer but larger clusters within the cell (Fig. 3F). Through N&B analysis, the cell edge showed a significant increase (80%) in the highest state of aggregation observed and a 63% increase in the average B compared to the control (Fig. 3A,B, triangles). Significant increases in aggregation were also determined in the cytoplasm and peri-nuclear areas, which demonstrated 70% and 97% increases in the average aggregation, respectively (Fig. 3B, triangles). Compared to the controls, these cells exhibited a decrease of about 50% in the particle number along the cell edge. The cytoplasm and peri-nuclear region however, showed increased averages of 16% and 49% compared to the controls, respectively (Fig. 3C, triangles). Supplementary Figure 2 presents the graphs of Fig. 3 with treatments plotted side-by-side with significant differences displayed between cell locations.

### DNA Size Influence on Lipoplex Aggregation

**Figure 4:**
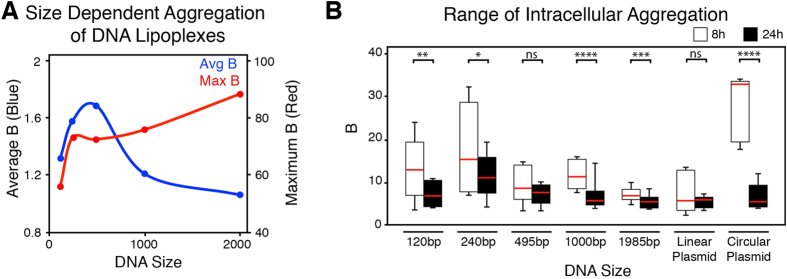
Influence of DNA Size on Lipoplex Aggregation. (**A**) Size-dependent aggregation of DNA lipoplexes formed in solution. Quantification of average (blue line) and maximum (red line) brightness using N&B methods. (**B**) 8 and 24 hours after delivery of various sized DNA fragments into myoblasts (n = 6 cells in each group). Box and whisker plots show the IQR with the whiskers providing the upper and lower ranges observed. The red line in the box represents the mean value. Statistical analysis between the different DNA sizes has been presented in Supplementary Figure 4. ns = no significantly difference, * p ≤ 0.05, ** p ≤ 0.01, *** p ≤ 0.001, **** p < 0.0001.

Figure 4 shows the effects of the size of DNA on aggregation using small (120 bp) to large (2 kbp) DNA fragments, as well as linear and circular plasmids (5.5 kbp). In solution, the smaller DNA fragments (i.e. 120 bp) aggregated less compared to the larger (2 kbp) fragments once in lipoplexes (Fig. 4A). Similarly to previously observed, as the aggregation of the lipoplex increased, the average B decreased.

Figure 4B shows the degree of lipoplex aggregation from different DNA lengths following transfection into the myoblast cells. Images were collected 8 and 24 h after the administration of the lipoplexes. Once within the cytoplasm the lipoplexes demonstrated a size dependent aggregation, which was in contrast to that in solution. The smaller DNA sizes exhibited greater aggregation than the larger. For example, the average maximum aggregative state in the 240 bp was 17.7, compared to 7.1 in the 1,984 bp at 8. Overtime, across all DNA sizes, the aggregation decreased between 20–75% (2 kbp and circular plasmid, respectively) with a mean decrease of 40%. At 24 h, most DNA fragments exhibited aggregative states that were statistically insignificant, in particular between the 1–5.5 kbp range (Supplementary Figure 4). Additionally, at 8 h, the circular plasmid exhibited a 3.5-fold difference in aggregation compared to the linear plasmid, demonstrating that DNA conformation plays a role in aggregation.

## Discussion

Here we have presented a method to quantify the aggregation of lipoplex delivered DNA in live cells through the application of the N&B approach. The N&B technique enables a non-invasive measurement of the molecular brightness relevant for aggregative states, as well as particle number, within a confocal image series. Through the combination of this approach, and the delivery of lipoplexes, we have been able to characterise the aggregation and particle number distribution within live cells, as well as explore the effects that serum and DNA size have to lipoplex aggregation. This study presents the first quantification of DNA aggregation, when delivered through lipofection in live cells.

By culturing the cells in different conditions, it has been shown that the cells respond and process the delivered DNA differently based on the conditions and status of the cell. It may also be possible that the extracellular serum interacts with the lipoplexes before or during internalisation, altering their chemistry. The N&B approach has demonstrated to discriminate between these changes and differences that have occurred.

Previous emphasis has been on the aggregation of DNA during complex formation and has mostly focused on the use of polyethylenimine (PEI)^17^,^18^. Such studies have identified that aggregation can be affected by factors such as pH and viscosity^17^. Additionally, previous studies have shown that aggregation occurs when lipoplexes are exposed to ionic conditions^19^, possibly a cause of aggregation within the cell. However, limited opportunities and approaches to address the aggregation of DNA delivery vectors in live cells have been a problem. Previously within live cells, it was found that a lack of serum resulted in less DNA clustering compared to cells maintained in 10% serum^20^. However, in these studies confocal microscopy was used to observed cluster size and did not quantify the extent of aggregation that had occurred.

The size dependent aggregation has been attributed to the charge of the DNA, given that shorter DNA fragments has a lower net charge compared to their larger counterparts. The formation of lipoplexes is a random event, which relies on the electrostatic interactions between the negatively charge DNA backbone and positively charged (cationic) lipids^1^. Therefore, it would appear that larger DNA fragments with a greater charge form larger lipoplexes.

As lipofection is a common practise in many cell biology-focused groups, the N&B approach offers an avenue to evaluate approaches, reagents and protocols when optimising transfections. The main requirements is that the DNA is fluorescently labelled, which can be achieved through a number of methods, and the N&B approach can be applied to many commercially available systems^11^. Also worth noting, this technique would also have the capacity to assess how much residual DNA-lipid complexes remain within the cell following an amount of time. If allowed to accumulate, the complexes would be expected to alter the biology of the cell, and may induce cytotoxicity.

Finally, the application of the N&B approach to determine DNA lipoplex aggregation offers a new focus of this type of bioimaging approach. In many previous applications, the N&B approach was applied to study the aggregation and behaviour of proteins tagged with a fluorescence protein, such as GFP, and genetically encoded. On the contrary, this study has utilised small molecular probes a fraction of the size of GFP and endeavoured to address the processing of an exogenous compound moving into and through the cell. Therefore, we hope that this study presents a model for new application of this bioimaging approach, as it is highly adaptable and could potentially be applied to address the delivery of other macromolecules, including drugs, nanoparticles and other nucleic acids.

Overall, our results show that delivered DNA facilitated by lipofection, aggregates to a large extent within the cell, and is influenced by a number of factors including the location, the environmental conditions such as serum concentrations, and the DNA itself. Our study was the first to quantify the extent of aggregation that occurs in delivered DNA showing differences and variation in aggregation across the cell. Now that a quantifiable approach has been established to study such a phenomena, it is imperative to further understand the mechanisms involved in the aggregation of DNA, and whether it is detrimental to gene delivery outcome as currently believed, or if the process is necessary.

## Methods

### Production of DNA Fragments

The PCI-Neo mammalian vector (Promega, Madison WI) was fluorescently labelled with Alexa Fluor 488 through a UYLSIS nucleic acid labelling kit (Molecular Probes, Eugene OR), briefly, the DNA plasmid was suspended in labelling buffer and denatured at 95 °C for 5 min then plunged in ice. Alexa Fluor 488 labelling reagent was dissolved in DMSO, and added to the DNA mixture at a 3:1 ratio (DNA: reagent). The labelling reaction was carried out at 80 °C for 15 min, before being stopped on ice and purified via a spin column (Qiagen). The labelled plasmid was either kept circular or linearized by digesting with BgI II (digestion was confirmed through agarose electrophoresis).

An Alexa Fluor488 labelled 21 bp oligonucleotide (5’-AF488-TCAATATTGGCCATTAGCCAT-3’) was synthesised by Integrated DNA Technologies (Coralville, IA) and used as a forward primer to produce 120, 240, 495, 1000 and 1985 bp fragments (reverse primers: 5’-GGACATGAGCCAATATAAATGTACA-3’, 5’-GGGCCATTTACCGTAAGTTATG-3’, 5’-ACGTAGATGTACTGCCAAGTAGGA-3’, 5’-GATGTCAGTAAGACCAATAGGTGC-3’, 5’-GTGTGAAATACCGCACAGATG-3’, respectively) using PCI-Neo plasmids as template. PCR reactions were performed using a Platinum Taq DNA polymerase (Invitrogen). DNA fragment lengths were confirmed through agarose electrophoresis.

### Cell Culture and Transfection

Rat L6 myoblast cells (ATCC CRL-1458) were maintained in DMEM (Invitrogen) supplemented with 10% (vol/vol) FBS (Invitrogen). In the study of FBS effects, cells were maintained in 2.5, 10 or 20% FBS 24 h prior to transfection, and then maintained in the same conditions throughout experimentation. Cells were transfected in 8-well glass chamber slides (Thermo Fisher Scientific, Australia) using X-Treme Gene 9 (Roche, Germany), according to the manufactures’ guidelines with a 1:3 DNA:lipid ratio. To prepare lipoplexes, (at room temperature) 50 μL of serum-free DMEM was combined with 0.45 μL of lipid reagent, followed by 150 ng of fluorescently labelled DNA. This mix was incubated for 15 min, and then added directly to cell preparations. For cytoplasmic measurements, the cells were imaged between 4–8 hr after introduction of the lipoplexes, whereas, nuclear measurements were imaged after 36 hr. Cell nuclei were counterstained with Hoechst 33342 (1 μg/mL).

### Solution Measurements

The Alexa Fluor488 labelled DNA was either kept naked, or lipoplexes were formed as above. In solution, 150 ng of naked or complexed DNA was diluted in 400 μL of serum free DMEM and imaged the same as the cells below at 37 °C.

### Image Acquisition and Data Analysis

Confocal images and N&B and RICS data was acquired using a Leica True Confocal Scanner – Spectro-Photometer 5 (TCS-SP5) inverted confocal microscope (Leica Microsystems, Germany) equipped with a 63 × 1.4 NA water immersion objective. N&B and RISC data was acquired using Avalanche Photodiodes (APDs) fitted with a 500–550 nm filter (Leica Microsystems, Germany). The TCS-SP5 was coupled and synchronised with an ISS Vista Becker and Hickl FCS card and software (Becker & Hickl GmbH, Germany), from which the N&B and RICS data were collected through. Confocal images were collected with two channels, 415–480 nm for Hoescht 33342 and 500–550 nm for Alexa Fluor488 labelled DNA. All data was collected in a 256 × 256 format with a pixel dwell speed of 32 μs and at 37 °C. All N&B and RICS data series were collected with 100 frames. 405 nm, 488 nm and 633 nm laser lines were activated for Hoescht 33342, Alexa Fluor488 and brightfield excitation, respectively. Throughout all experiments the 488 nm Argon laser line was used at the same power so that experiments can be compared to each other. Altering the laser power would result in different brightness value being obtained, and therefore experiments would not be comparable.

N&B and RICS data were analysed using the Globals software package, SimFCS 2.0, developed at the Laboratory for Fluorescence Dynamics at the University of California, Irvine (www.lfd.uci.edu/globals). The waist of the PSF was determined with eGFP as previously described for the RICS analysis^21^. All statistical analyses were performed with GraphPad Prism 6 software using an unpaired, two-tailed Student’s t-test, with data presented as mean ± se.

## Additional Information

**How to cite this article**: Mieruszynski, S. *et al.* Live Cell Characterization of DNA Aggregation Delivered through Lipofection. *Sci. Rep.* **5**, 10528; doi: 10.1038/srep10528 (2015).

## Supplementary Material

Supplementary Information
