# Supplementary material for: Live Cell Characterization of DNA Aggregation Delivered through Lipofection
Source: Sci Rep. 2015 May 27;5:10528. doi: 10.1038/srep10528 (PMC4444954; doi:10.1038/srep10528)
Supplement: Supplementary Information [file srep10528-s1.pdf]

Live Cell Characterization of DNA Aggregation Delivered through Lipofection

Stephen Mieruszynski [¶], Candida Briggs [¶], Michelle A. Digman [§], Enrico Gratton [§], Mark R Jones [¶*]

Author affiliation:

[¶] University of Western Sydney, School of Science and Health, Hawkesbury Campus, Locked Bag 1797, Penrith NSW 2751, Australia

[§] Department of Developmental and Cell Biology, University of California Irvine, Irvine, California, United States of America; Department of Biomedical Engineering, Laboratory for Fluorescence Dynamics, University of California Irvine, Irvine, California, United States of America.

[§] Centre for Bioactive Discovery in Health and Ageing, School of Science & Technology, University of New England, Armidale, Australia.

** Corresponding author*

Supplementary Figure 1. ROI analysis of DNA Aggregation

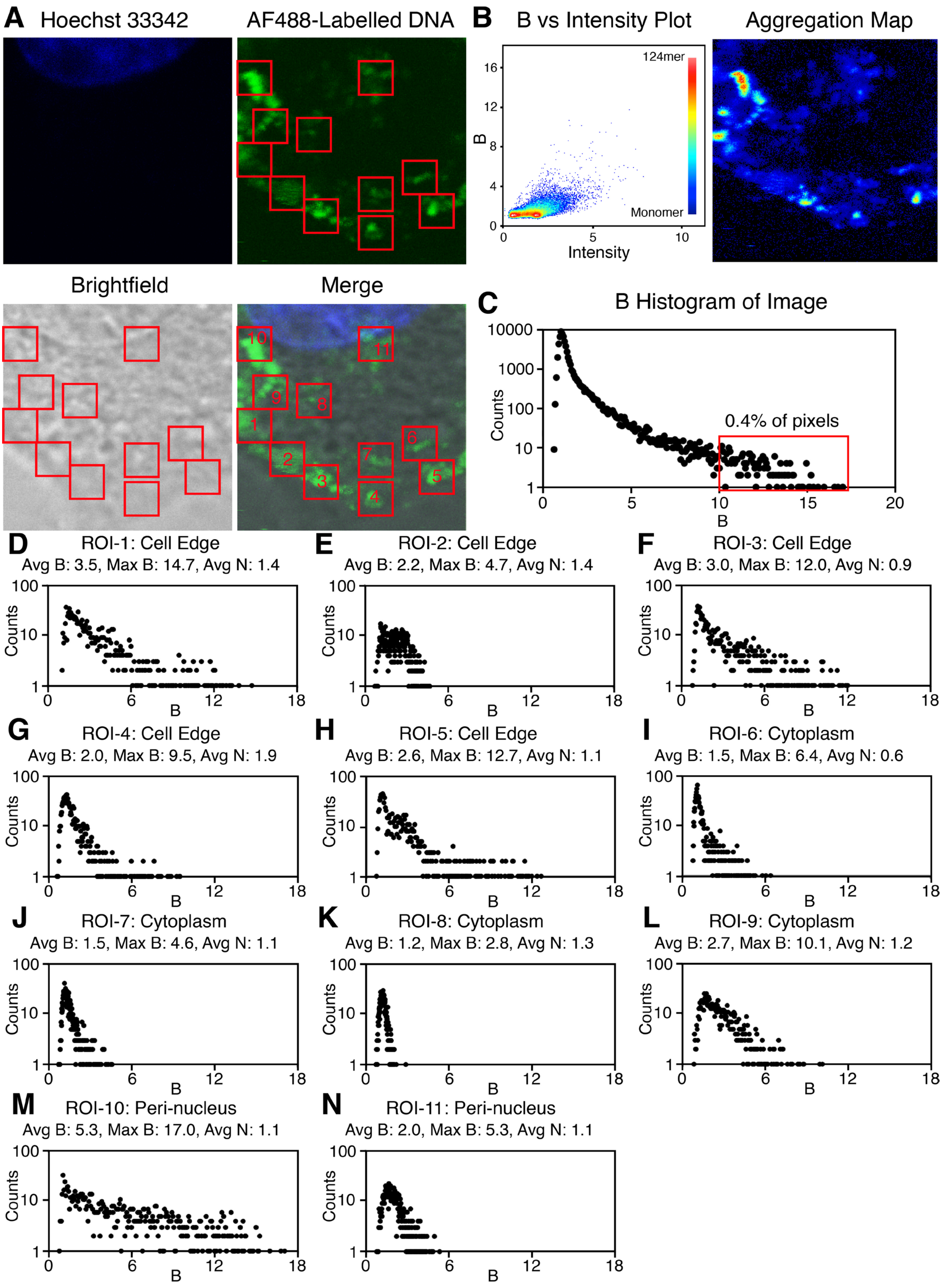

Supplementary Figure 1. ROI analysis of cell presented in Figure 2A from main article. (A) Confocal images of cell transfected with fluorescent DNA plasmid, and imaged 6h after

administration of lipoplexes. Confocal images depict the nucleus (blue, Hoechst 33342), fluorescently-labelled DNA (green), brightfield (grey) and overlay of channels. ROIs were selected and displayed over the images. (B) N&B analysis of cell, displaying the localization of DNA aggregates. (C) B histogram of entire cell. The B values > B = 10 have been boxed, accounting for 0.4% of pixels in the displayed images. (D-N) The average B, maximum B, average N and B histogram of ROIs 1-11, highlighted in image A. ROIs represent areas of the cell including the cell edge (ROIs 1-5), cytoplasm (ROIs 6-9) and peri-nuclear region (ROIs 10-11).

Supplementary Figure 2. Effects of Serum Concentrations on DNA Aggregation

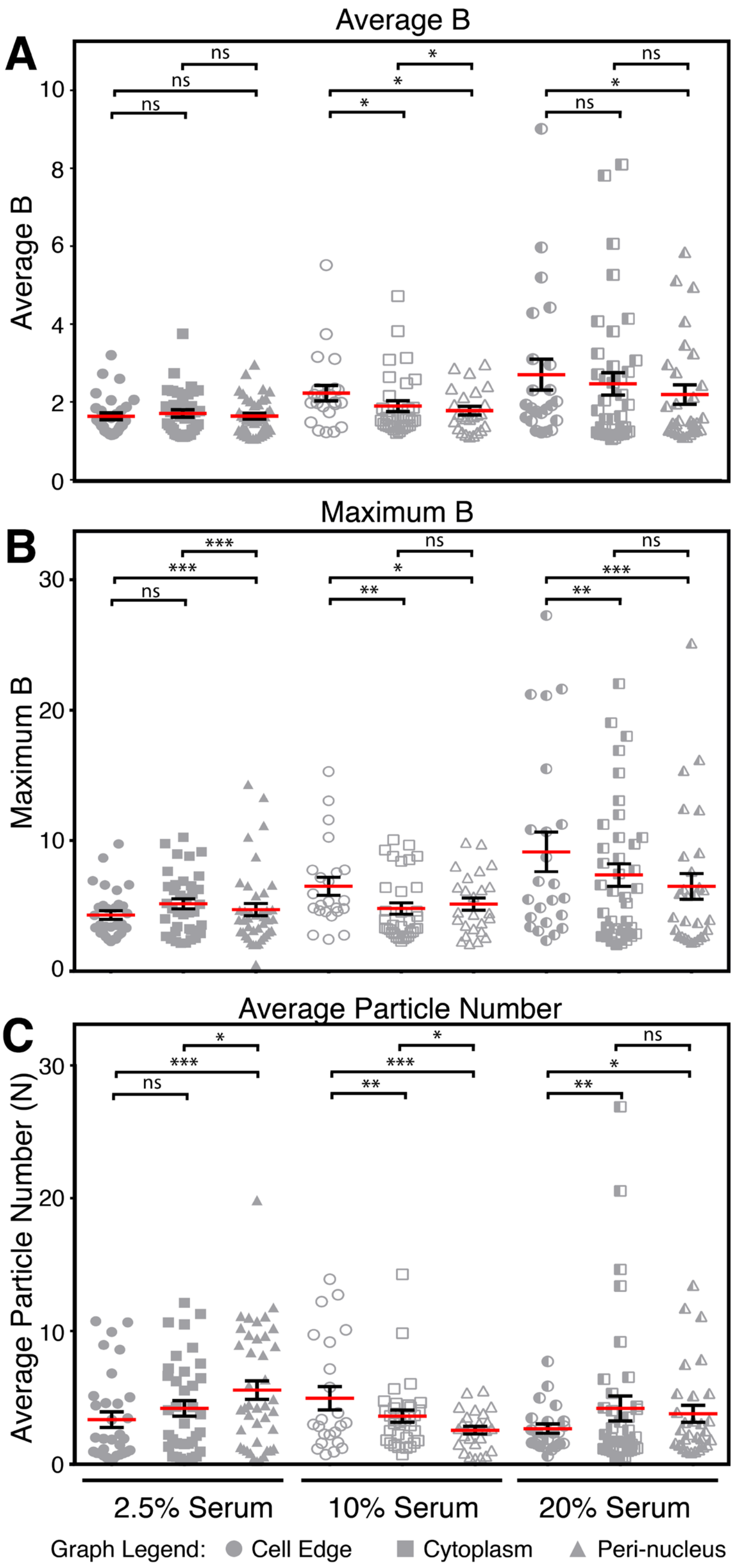

Supplementary Figure 2. Effects of serum of DNA aggregation and particle number. ROI analysis of DNA aggregation and particle number of cells maintained in 2.5% (full markers), 10% (empty markers) and 20% (half markers) serum. Data has been plotted showing the comparison of the cell edge (circle), cytoplasm (square) and peri-nuclear region (triangle) in each serum concentration. Graphs depict mean ± se. ns = no significantly difference, * $p \leq 0.05$, ** $p \leq 0.01$, *** $p \leq 0.001$, **** $p < 0.0001$.

Supplementary Figure 3 – B Histogram of cells maintained in different serum concentrations

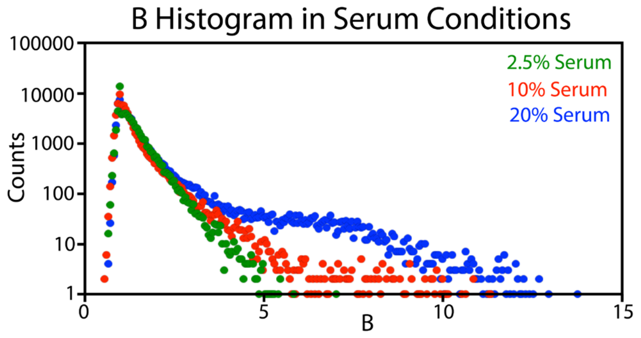

Supplementary Figure 3. B histogram of cells presented in Figure 3D-F. B Histogram depicts differences in aggregation when cells are maintained in different serum concentrations, including 2.5% (green), 10% (red) and 20% (blue) serum. Cells maintained in 2.5% serum demonstrate the least amount of aggregation, and those maintained in 20% serum had the greatest extent of aggregation.

Supplementary Figure 4 – Statistical analysis of aggregation due to DNA size

8 Hour

	Circular 5.5kbp	Linear 5.5kbp	1,985	1,000	495	240
120	****	**	***	*	**	ns
240	***	***	***	ns	***	
495	****	ns	**	**		
1,000	****	***	****			
1,985	****	*				
Linear	****					

24 Hour

	Circular 5.5kbp	Linear 5.5kbp	1,985	1,000	495	240
120	ns	**	ns	ns	ns	**
240	***	****	***	***	**	
495	**	**	**	**		
1,000	ns	ns	ns			
1,985	ns	ns				
Linear	ns					

* $p \leq 0.05$

** $p \leq 0.01$

*** $p \leq 0.001$

**** $p \leq 0.0001$

ns = no significant difference

Supplementary Figure 4. Significant differences in aggregation from DNA of various sizes at 8 and 24h. ns = no significantly difference, * $p \leq 0.05$, ** $p \leq 0.01$, *** $p \leq 0.001$, **** $p < 0.0001$.
